# MHC Class I Presented T Cell Epitopes as Potential Antigens for Therapeutic Vaccine against HBV Chronic Infection

**DOI:** 10.1155/2014/860562

**Published:** 2014-05-26

**Authors:** Joseph D. Comber, Aykan Karabudak, Vivekananda Shetty, James S. Testa, Xiaofang Huang, Ramila Philip

**Affiliations:** ^1^Immunotope, Inc., Doylestown, PA 18902, USA; ^2^Baylor College of Medicine, Houston, TX 77030, USA; ^3^Celldex Therapeutics, Hampton, NJ 08827, USA

## Abstract

Approximately 370 million people worldwide are chronically infected with hepatitis B virus (HBV). Despite the success of the prophylactic HBV vaccine, no therapeutic vaccine or other immunotherapy modality is available for treatment of chronically infected individuals. Clearance of HBV depends on robust, sustained CD8^+^ T activity; however, the limited numbers of therapeutic vaccines tested have not induced such a response. Most of these vaccines have relied on peptide prediction algorithms to identify MHC-I epitopes or characterization of T cell responses during acute infection. Here, we took an immunoproteomic approach to characterize MHC-I restricted epitopes from cells chronically infected with HBV and therefore more likely to represent the true targets of CD8^+^ T cells during chronic infection. In this study, we identified eight novel MHC-I restricted epitopes derived from a broad range of HBV proteins that were capable of activating CD8^+^ T cells. Furthermore, five of the eight epitopes were able to bind HLA-A2 and A24 alleles and activated HBV specific T cell responses. These epitopes also have potential as new tools to characterize T cell immunity in chronic HBV infection and may serve as candidate antigens for a therapeutic vaccine against HBV infection.

## 1. Introduction

Hepatitis B virus (HBV) is a member of the Hepadnaviridae family of viruses which also includes woodchuck hepatitis virus (WHV) and duck hepatitis B virus. These viruses are primarily hepatotropic with infections characterized by fever, fatigue, muscle aches, and yellowing of the eyes and/or skin. The severity of these symptoms can vary with a proportion of cases being asymptomatic [1]. More than 2.5 billion people worldwide have been infected by HBV, but, for the vast majority of adults encountering the virus (>90%), the infection is acute and readily cleared by the immune system [2, 3]. For the remaining 5–10% of adults, and for neonates and unvaccinated children, HBV establishes a chronic infection. Approximately 370 million people worldwide are chronically infected and over 500,000 people die each year due to complications from HBV [1, 4]. These complications include liver cirrhosis, liver failure, and/or hepatocellular carcinoma (HCC) and it is estimated that up to 40% of chronically infected patients will develop at least one of these complications [5].

The primary determinant of whether hepatitis B virus is cleared or establishes a chronic infection is the robustness of the immune response, in particular the CD8^+^ T cell response [6–9]. Data from both animal models and infected patients indicate that strong innate immune responses are crucial in controlling initial HBV replication and for subsequently activating the adaptive T cell response (reviewed in [2, 3]). In patients that resolve acute infections, there are greater numbers of IFN-*γ* secreting CD4^+^ and CD8^+^ T cells [10] with a broader range of epitope recognition [11, 12] than in chronically infected patients [3, 13]. Although individuals that initially fail to mount vigorous T cell responses develop chronic infection, data indicate that virus specific T cells are still capable of a broad, effective T cell response. Rehermann et al. demonstrated that a small number of chronically infected individuals mount robust CTL responses against HBV either spontaneously or in response to IFN-*α* treatment [14]. These T cells are directed against multiple proteins indicating that chronically infected patients can also mount a broad response to viral antigens. These data suggest that therapeutic interventions designed to stimulate robust and multiepitope specific responses may be sufficient to resolve chronic HBV infections. Yet, despite an effective prophylactic vaccine, there are currently no therapies capable of eliminating HBV from chronically infected individuals. A number of anti-HBV therapeutic vaccines have been tested including traditional prophylactic vaccines, antigen/antibody complexes [15], lipopeptide [16], DNA [17], and recombinant virus [18] based strategies with limited success. Thus, there is a critical need for more targeted therapeutic vaccines capable of inducing robust, sustained T cell responses capable of permanent clearance of virus.

Therapeutic peptide based vaccines are an attractive method for inducing CD4^+^ and CD8^+^ T cell responses in chronically infected individuals. Formulating a vaccine with multiple epitopes presented by the chronically infected cells that are capable of activating polyclonal T cell responses may have the ability to eradicate the infected cells in chronically infected patients. Peptide antigens for the early stage clinical studies were identified by motif prediction algorithms and selected by screening CTLs from acute and chronically HBV infected patients [19]. However, the T cell epitopes presented by HBV infected cells have not been reported or used in a clinical study. Here we took an immunoproteomic approach to identify MHC class I peptides presented by chronically HBV infected cells. This approach has distinct advantages over traditional vaccine design algorithms as it identifies antigens naturally processed and presented by infected, but not healthy, cells. Using this approach we identified 8 naturally processed HLA-A2 restricted epitopes capable of stimulating robust CD8^+^ T cell responses. Interestingly a subset of these epitopes is also capable of binding HLA-A24 molecules and stimulates both HLA-A2 and HLA-A24 restricted T cell responses.

## 2. Materials and Methods

### 2.1. Mice

Four- to eight-week-old female HLA-A2 transgenic (CB6F1-Tg(HLA-A*0201/H2-K^b^)A*0201) or HLA-A24 transgenic (CB6F1-Tg(HLA-A*2402/H2-K^b^)A24.01) mice were obtained from Taconic and housed at Lampire Biologicals (Pipersville, PA). All animal experiments were conducted in adherence to the Guide for Care and use of Laboratory Animals of the NIH. Experimental protocols were approved by the Institutional Animal Care and Use Committee of Lampire Biologicals.

### 2.2. Cells, Viruses, and Plasmids

The HLA-A2 and A24 positive liver hepatocellular carcinoma cell line HepG2 and its hepatitis B infected derivatives HepDE19 and HepG2.2.15 were cultured in Dulbecco's Modified Eagle Medium/Ham's F-12 50/50 Mix (Mediatech Inc, Manassas VA). 293-T cells were maintained in Dulbecco's' Modified Eagle Medium and T2 cells were maintained in RPMI-1640 (Mediatech Inc). All media were supplemented with 10% fetal bovine serum (Atlanta Biologicals, Flowery Branch, GA), L-glutamine (300 mg/mL), 1x nonessential amino acids, 0.5 mM sodium pyruvate, and 1x penicillin/streptomycin (Mediatech Inc). Cells were maintained at 37°C and 5% CO_2_.

Adenovirus containing the HBV genome (Ad-HBV) was a kind gift of Dr. Anand Mehta (Drexel University). 293T-HLA.A2^+^ cells were seeded into 6-well plates and infected with AdHBV virus. 48–72 hours later, the cells were harvested and used in downstream applications.

### 2.3. Isolation, Purification, and Fractionation of MHC Class I Bound Peptides

Hepatitis B virus specific MHC class I restricted peptides were isolated as previously described [20]. Briefly, 1 × 10^9^ HepDE19 cells were lysed (150 mM NaCl, 10 mM Na_2_HPO_4_, 1 mM EDTA, 1%NP40) and peptide/MHC complexes (p/MHC) were immunoprecipitated using protein A/G beads (UltraLink Immobilized Protein A/G, Pierce, Rockford, IL) coated with W6/32, a monoclonal antibody that recognizes pan-MHC class I molecules. Antibody coated beads were incubated with cell lysate for 2 hours with rocking and then separated from the lysate by centrifugation (1000 rpm/5 min). The p/MHC complexes were eluted from the beads using 0.1% trifluoroacetic acid and the peptides were dissociated from the MHC molecules by heating at 85°C for 15 minutes. The mixture was cooled and the peptides separated using an Amicon Ultra-3 kDA filter (Millipore, Billerica, MA). The resulting peptide mixture was then fractionated using a C-18 reversed phase (RP) column on an offline 3000 HPLC (Dionex).

### 2.4. Mass Spectrometry Analysis

Mass spectrometry experiments were carried out using Orbitrap instruments (Thermo Electron, San Jose, CA) interfaced with nano ultimate high-performance liquid chromatography (HPLC; Dionex). RP-HPLC-purified peptide fractions were injected individually into the LC-MS/MS system to identify the sequences [20, 21] of the peptides. The peptides were concentrated using a 300 *μ*m ID × 5 mm C18 RP trap column (Dionex) and separated using a 75 *μ*m ID × 15 cm C18 RP analytical column (Dionex), equilibrated in 4% ACN/0.1% FA at 250 nL/minute flow rate. Mobile phase A was 2% ACN and 0.1% FA in water, whereas mobile phase B was 0.1% FA and 90% ACN in water. Peptides were separated with a gradient of 4%–50% B in 60 minutes and 50%–80% in 90 minutes and eluted directly into the mass spectrometer. Peptides were analyzed using a data-dependent method. The acquired spectra data were searched against HBV protein database using Proteome Discoverer 1.3 (Thermo) to interpret data and derive peptide sequences. The database search parameters were enzyme-no enzyme, threshold-100, peptide tolerance-20 ppm, and fragment ion tolerance-0.8 Da. The search results were filtered with XCorr according to individual peptide charge status (+1 : 1.6, +2 : 1.8, and +3 : 2.0) and the results were also verified manually to confirm the correct peptide sequence.

### 2.5. Peptide Validation by Synthetic Peptides

Synthetic peptides for validating the peptides identified in this study were obtained from GenScript Corporation and China peptides Co., Ltd. The synthetic peptides were subjected to LC-MS/MS analysis under identical experimental conditions as described above, and their sequences were confirmed based on their MS/MS data. Candidate peptide sequences were confirmed by comparison of MS/MS spectra with synthetic analogues.

### 2.6. Generation of Epitope Specific CTLs* In Vitro*

Peptide specific CTLs were generated as previously described [20–23]. Briefly, PBMCs were isolated from heparinized blood of healthy HLA-A2^+^ donors (Research Blood Components, LLC, Boston MA) using lymphocyte separation medium (Corning, Corning, NY) and cultured in 6-well plates overnight in RPMI-1640. The next day, the nonadherent cells were harvested and saved and the adherent cells were pulsed with MHC class I restricted synthetic peptides (50 *μ*g) + *β*2 microglobulin (1.5 *μ*g) in order to selectively expand epitope specific CD8^+^ T lymphocytes (CTLs). After a two-hour incubation, the nonadherent cells were resuspended in a cytokine rich medium of RPMI-1640 supplemented with IL-7 (5 ng/mL), GM-CSF (25 ng/mL), IL-4 (50 ng/mL), and keyhole limpet hemocyanin (5 ug/mL KLH; Sigma-Aldrich). The nonadherent cells were added back into the 6-well plates to a final volume of 5 mL/well and cultured at 37°C at 5% CO_2_. T cells in culture were restimulated 12–14 days after initial stimulation with autologous PBMCs depleted of CD4^+^ and CD8^+^ T cells and pulsed with synthetic peptides (10 ug/mL) and *β*2-microglobulin (1.5 ug/mL). Restimulated cells were cultured in RPMI-1640 supplemented with IL-15 (5 ng/mL), GM-CSF (12.5 ng/mL), and IL-2 (10U/mL) for 7 days. Restimulation was performed a total of three times prior to CTL functional assays. Unless otherwise specified, all cytokines and growth factors were purchased from eBiosciences (San Diego, CA).

### 2.7. Generation of Epitope Specific CTLs* In Vivo*

All transgenic mouse manipulations (i.e., injections and spleen harvests) were carried out at Lampire Biologicals. HLA-A2^+^ and HLA-A24^+^ transgenic mice were injected with PBS alone or 10 ug of synthetic peptides in PBS or a 50 : 50 emulsion with Montanide ISV 51 (Seppic Inc, Fairfield, NJ). Mice were injected at two sites: subcutaneously (s.c.) on the flank and intradermally (i.d.) near the base of the tail. Mice received a total of three injections, at 10 days intervals. A week after the third injection, mice were euthanized and the spleens were harvested for use in T cell functional assays.

### 2.8. ELISpot Assays

96-well PVDF-membrane plates (Millipore) were coated with IFN-gamma capture antibody overnight at 4°C. On the day of the assay, the plates were blocked for 2 hours in RPMI-1640 complete medium and washed prior to use in the ELISpot assay.* In vitro* generated CTLs were assayed 7 days after the final restimulation. Peptide specific CTLs were harvested, counted, and cultured overnight with appropriate antigen presenting cells that were unpulsed or pulsed with synthetic peptides (T2 or HepG2 cells) or antigen presenting cells that are productively infected with HBV (HepDE19, HepG2.2.15, and 293/T/A2 Ad-HBV infected cells). The next day the assay was developed according to the manufacturer's instructions (BD Biosciences, San Jose, CA).* In vivo* generated CTLs were assayed 7 days after the final peptide injection. Spleens were harvested and homogenized into a single cell suspension. After lysis of RBCs, the cells were extensively washed, counted, and cultured overnight with HepG2 cells unpulsed or pulsed with synthetic peptides or HepDE19 cells. The next day the assay was developed according to the manufacturer's instructions (BD Biosciences). For both ELISpot assays, spots were quantified using the ELISpot Reader System (AID, San Diego, CA).

### 2.9. MAGPIX Cytokine Detection

Cytokine secretion from activated CD8^+^ T cells was measured using a customized MILLIPLEX magnetic bead assay according to manufacturer's instructions (Millipore). Briefly, supernatants were harvested from CD8^+^ T cells stimulated with various targets and cleared of cellular debris by a brief centrifugation. 25 *μ*L of samples, standards, and controls was added to a 96-well plate containing assay buffer (1 : 1 dilution). Next, magnetic beads coated with antibodies against the cytokines being analyzed were added to each well. The plate was then sealed and incubated on a plate shaker overnight at 4°C. The next day, the plate was washed twice with wash buffer and biotinylated detection antibodies were added. The plate was sealed again and rocked for 1 hour at RT followed by the addition of streptavidin-PE for additional 30 minutes. The plate was washed twice with wash buffer, loaded with sheath fluid, and read on the MAGPIX system. Data was analyzed with Milliplex Analyst software according to manufacturer's instructions (Luminex, Austin, TX).

### 2.10. Flow Cytometry Analysis

To detect epitope specific, cytotoxic CD8^+^ T cells (CD8^+^CD107a^+^), splenocytes derived from peptide primed mice were cultured with HepG2 cells left unpulsed or pulsed with specific peptides or with HepDE19 cells for six hours in the presence of anti-CD107a-PE (BD Biosciences). After six hours, cells were washed and CD8^+^ T cells were detected by addition of anti-CD8*α*-FITC (eBiosciences). For verification of infectivity, HepG2, HepDE19, HepG2.2.15, or 293T/A2/HBV cells were washed, fixed, and permeabilized with the Cytofix/Cytoperm kit according to manufacturer's instructions (BD Biosciences). Cells were incubated with antibodies against HBV core (Abcam, Cambridge, MA) or HBV sAg (Santa Cruz Biotechnology, Dallas, TX) for 30 minutes at RT. Following extensive washes with permeabilization wash buffer, cells were incubated with anti-mouse IgG FITC conjugated secondary antibody (BD Biosciences) for 20 minutes at RT. Cells were washed extensively and resuspended in PBS/0.1% BSA before acquiring. All flow cytometry events were acquired on the Guava easyCyte 8HT (Millipore). Data was analyzed using InSight software on the guavaSoft 2.6 platform (Millipore).

## 3. Results

### 3.1. Identification of MHC Class I Presented Epitopes from Hepatitis B Virus Infected Cells by Nano LC/MS/MS Methods

**Figure 1:**
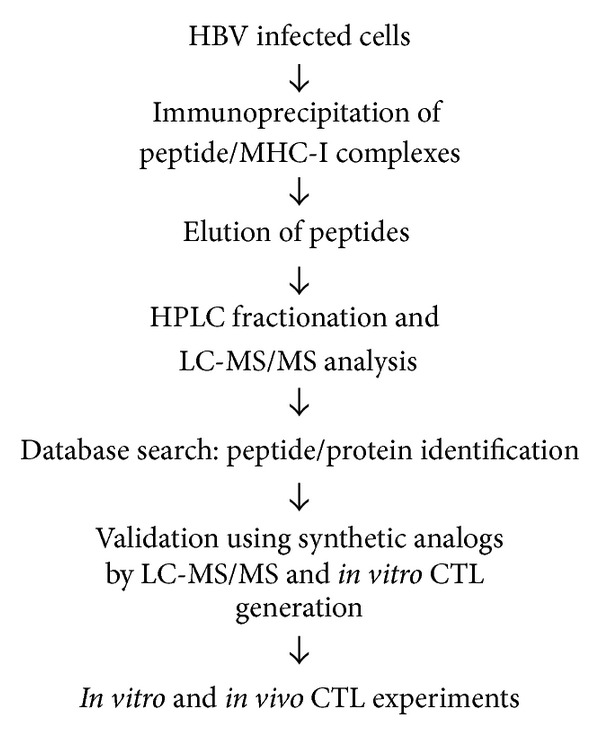
Immunoproteomics method work flow for the identification and characterization of HBV specific T cell epitopes.

**Figure 2:**
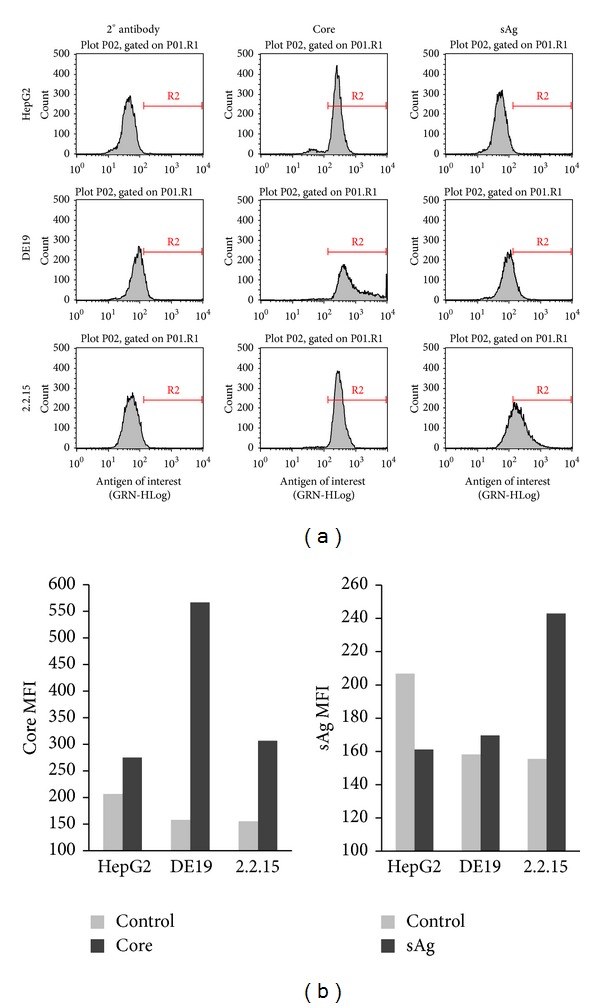
HBV protein expression in infected cell lines. (a) HepG2, DE19 (lacks sAg), and 2.2.15 (complete HBV genome) were harvested, fixed and permeabilized, and stained with HBV specific antibodies directed against core or sAg. (b) Median fluorescent intensity (MFI) derived from the flow plots shown in (a).

**Figure 3:**
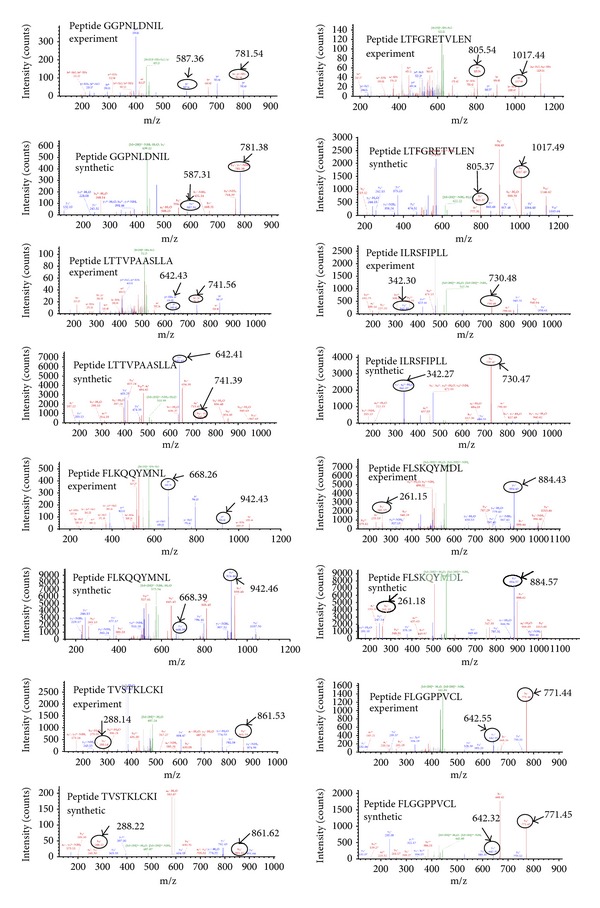
Validation of naturally presented MHC peptides from HBV infected cells. Mass spectrometry (MS/MS) spectra of the experimentally identified peptides (top spectra) versus their synthetic analogs (bottom spectra). Fragment masses that match are denoted.

T cell therapeutic vaccines are an attractive treatment option for individuals chronically infected with hepatitis B virus. In order for a therapeutic vaccine to induce sustained T cell responses, it must include antigens that are naturally processed and presented by the infected cells. Therefore, we set out to identify naturally processed and presented HBV specific MHC class I restricted epitopes using an immunoproteomics approach. In this approach, peptides associated with MHC class I molecules are isolated from the infected cells and identified using mass spectrometry analysis [20–27] (Figure 1). Peptide/MHC complexes were isolated from cells chronically expressing HBV antigens (HepDE19 and HepG2.2.15) and cells infected with an adenovirus encoding for the HBV genome (293T/A2-AdHBV). Using multiple cell types was essential because it allowed for identification of epitopes from the complete genome (e.g., HepDE19 do not express surface Ag (sAg)) (Figure 2). Using stringent mass spectra search criteria, we identified eight novel MHC-I peptide epitopes with high confidence: three HLA-A2 restricted peptides (GGP; LTF; LTT) and five peptides (ILR; FLK; FLS; TVS; FLG) that show promiscuity to binding both HLA-A2 and HLA-A24 (Table 1). The HLA binding affinities of the peptides calculated using the SYFPEITHI algorithm ([28] accessed via http://www.syfpeithi.de/) showed variable binding scores (Table 1). We then confirmed the sequence identity of these epitopes using synthetic analogs. The MS/MS spectra of the synthetic epitopes matched the spectra of the experimentally identified epitopes nearly identically (Figure 3). In addition to these novel epitopes, we identified 19 MHC-I restricted peptides derived from HBV that have been previously described by motif prediction method with low mass spec Xcorr search criteria that do not meet our established standards for high abundance and confidence and therefore were not included in our assays (see Table 1 in Supplementary Materials available online at http://dx.doi.org/10.1155/2014/860562).

### 3.2. Epitopes Identified by Immunoproteomics Analysis Activate HBV Specific CTLs* In Vitro*

**Figure 4:**
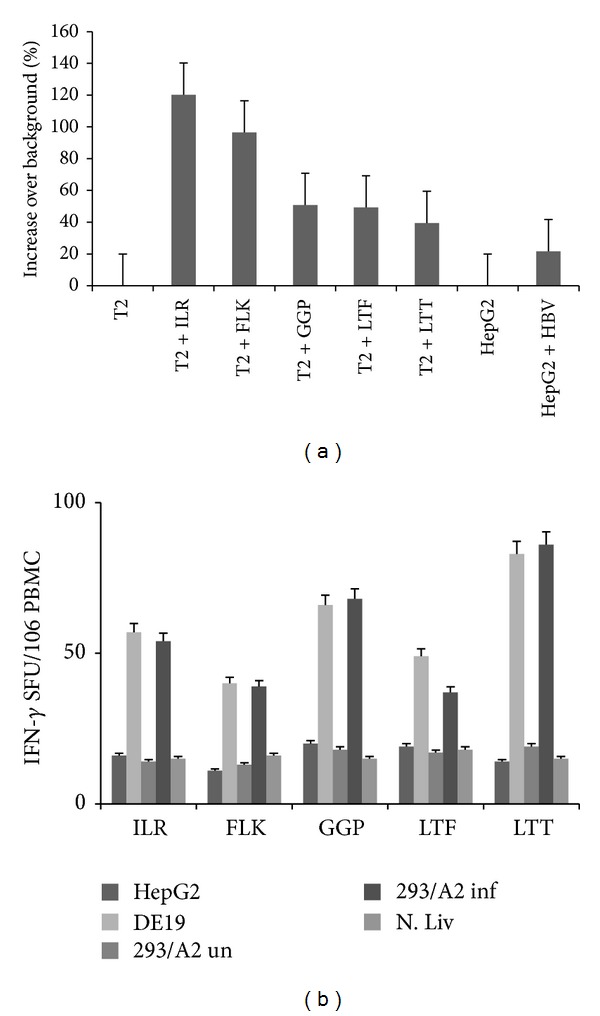
HBV specific peptides stimulate CD8^+^ T cell activation* in vitro*. (a) HLA-A2 restricted CTLs directed against the identified peptides (peptides are represented as first 3 residues of the sequence) were generated using peripheral blood from healthy donors. PBMCs containing the epitope specific CTLs were harvested, washed, and cultured with the peptide pulsed or HBV expressing cells overnight in an IFN-gamma ELISpot assay. Data is represented as % increase over background. (b) PBMCs containing epitope specific CTLs were harvested, washed, and cultured with uninfected or HBV expressing cells overnight in an IFN-gamma ELISpot assay. Normal liver cells served as a negative, nonspecific control.

After confirming the sequence of the experimentally identified epitopes, we determined if these epitopes could activate CD8^+^ T cells. To do so, we generated epitope specific CTLs from PBMCs isolated from healthy HLA-A2^+^ donors using the synthetic peptide versions. Epitope specific CD8^+^ T cells were activated (as measured by IFN-gamma ELISpot) when cultured with T2s pulsed with peptide (Figure 4(a)) and with HepDE19 or 293-T/A2-AdHBV cell lines (Figure 4(b)). Importantly, these responses were specific as only background CD8^+^ T cell activation was observed when the cells were cultured with normal liver, uninfected HepG2, or uninfected 293T/A2 cells (Figure 4(b)). Furthermore, the CTL responses did not correlate with their HLA binding affinities (Table 1).

### 3.3. Epitopes Identified by Immunoproteomics Analysis Activate HBV Specific CTLs* In Vivo*

**Figure 5:**
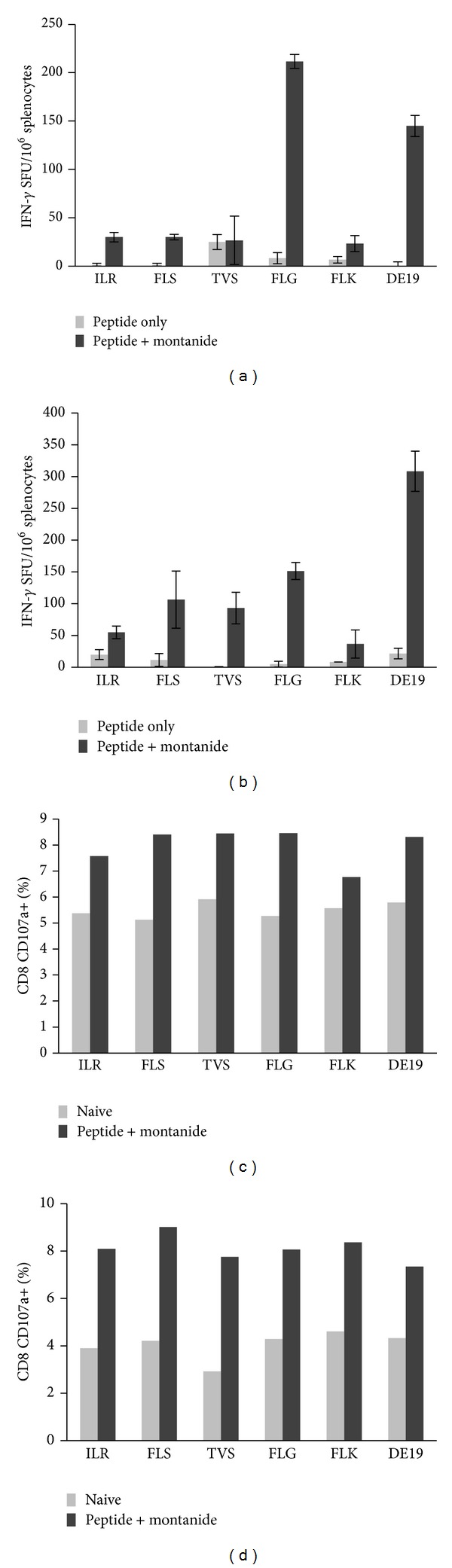
HBV specific peptides are able to activate CD8^+^ T cells* in vivo* in both an HLA-A2 and HLA-A24 restricted fashion. HLA-A2 (a) or HLA-A24 (b) transgenic mice were primed and boosted with peptides as previously described. Spleens were harvested, homogenized into single cell suspensions, and cultured with peptide pulsed (peptides are represented as first 3 residues of the sequence) HepG2 cells or HBV expressing cells overnight in an IFN-gamma ELISpot assay. T cell activation was also measured by examining CD107a upregulation on HLA-A2 (c) or HLA-A24 (d) CD8^+^ T cells. Splenocytes were cultured for 6 hours with peptide pulsed or HBV expressing cells in the presence of anti-CD107a and subsequently stained for CD8^+^ expression. Data is presented as the percent of cells in culture that are CD8^+^ CD107a^+^.

After establishing that epitopes could specifically induce CD8^+^ T cell activation* in vitro*, we next sought to determine if the experimentally identified peptides could also stimulate CD8^+^ T cells* in vivo*. Because a subset of our peptides (ILR; FLK; FLS; TVS; FLG) had the motif to bind both HLA-A2 and HLA-A24 molecules, we assessed CD8^+^ T cell activation of these peptides in both HLA contexts. Synthetic versions of these peptides were injected into HLA-A2^+^ or HLA-A24^+^ transgenic mice with or without Montanide ISV-51 adjuvant, three times in total. One week after the third injection, splenocytes were harvested and cultured with HepG2 cells pulsed individually with peptides alone or HBV infected cells in an IFN-gamma ELISPot assay. As shown in Figure 5(a), CD8^+^ T cells generated* in vivo *specifically recognized peptide loaded HepG2 cells as well as HBV infected HepDE19 cells. In addition, CD8^+^ T cells generated* in vivo* also upregulated a classical marker of degranulation (CD107a) [29, 30] after stimulation with both peptide pulsed HepG2s and HBV chronically infected cell line HepDE19 (Figure 5(b)). Interestingly, the peptides induced IFN-gamma secretion and CD107a upregulation independent of the HLA molecule tested which indicates that these HLA-A2 and HLA-A24 double binding peptides are capable of activating both HLA-A2 and HLA-A24 specific T cell responses.

**Figure 6:**
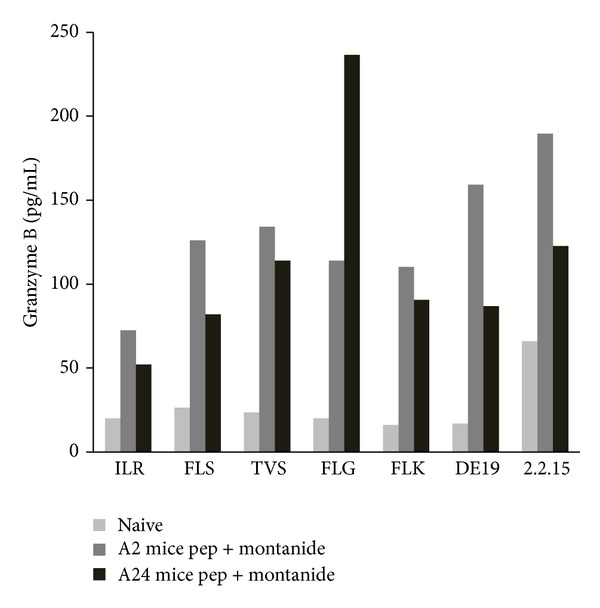
HBV peptide specific CD8^+^ T cells activated* in vivo* secrete cytotoxic effector molecules. In an assay that mirrored the setup described in Figure 5, splenocytes were cultured with peptide pulsed (peptides are represented as first 3 residues of the sequence) or HBV expressing cells overnight. Supernatant was harvested and used in the Milliplex magnetic bead assay to detect granzyme B secretion in response to specific stimulation. Splenocytes from PBS primed, naïve mice were used as a negative control.

Because degranulation is associated with delivery of perforin and granzyme to target cells, we next checked the levels of granzyme B being secreted by the peptide activated CD8^+^ T cells. Supernatants were collected from the stimulated cells and the levels of granzyme B were detected using Milliplex magnetic bead technology [31, 32]. CD8^+^ T cells from both HLA-A2 and HLA-A24 immunized mice secreted high levels of granzyme-B in response to peptide stimulation and HBV chronically infected HepDE19 cell stimulation compared to their naïve counterparts indicating the activation of a specific cytotoxic response (Figure 6).

## 4. Discussion

The peptides described in this report are newly identified epitopes and, to our knowledge, have not been reported elsewhere. Importantly, these peptides were derived from multiple viral proteins, thereby potentially increasing the targets for T cells and inducing the broad response needed for chronic viral clearance. Two peptides (GGP and LTT) are derived from the large E protein, one (LTF) is derived from the core protein, two (ILR and FLG) are derived from surface protein, and three (FLK; FLS; TVS) are derived from the polymerase protein. The diversity of our identified peptides is similar to that of the response induced during a natural infection (reviewed in [3]). Previous studies in HLA-A2 restricted models have identified immunodominant epitopes from core protein (HBc18-27; [11, 19]), envelope and polymerase proteins (HBe 348–357, HBp455-463; [19]), and X protein (HBx4-10; [33]). Although we were able to detect a few of these peptides in our analysis (including the clinically well studied HBcAg 18–27 epitope, FLPSDFFPSV), the scores (Xcorr values) obtained from MS/MS analysis did not meet standard abundance and confidence cutoff criteria for inclusion in our assays (Supplementary Table 1). A few potential explanations exist to clarify this observation. First, although a subset of these peptides was shown to induce T cell activation in patients acutely infected with HBV [19], T cell responses generated during acute HBV infections may very well differ from those necessary to clear virus during a chronic infection due to alterations in MHC peptide repertoire on chronically infected cells. Differences in epitope specificity between acute and chronic phases of infection have been observed in other models of chronic infections, for example, LCMV [34, 35] and HIV-1 [36]. Secondly, the low confidence epitopes identified may not be presented at very high levels on the surface of the chronically infected cells, which would lead to a low representation in the MS/MS analysis. Interestingly, several recent reports have demonstrated that low level antigen presentation preferentially favors the expansion of naïve but not memory CD8^+^ T cells [37–39], which may explain the transient (not sustained) increases in these epitopes specific CD8^+^ T cell responses in previous clinical trials. Together these data indicate that low scoring peptides may not be the right target for inclusion in therapeutic vaccines and the most relevant targets should be identified by analyzing chronically infected cells.

In order to identify epitopes that are naturally processed and presented by chronically infected cells, we utilized an immunoproteomic approach. Using this approach, we identified eight novel MHC class I restricted epitopes that are derived from four different proteins of the HBV genome. We first confirmed that the identified peptides were able to induce CD8^+^ T cell activation by measuring IFN-gamma production in an ELISpot assay. Importantly, the T cells were able to recognize the naturally processed and presented epitope appearing on a variety of HBV infected cells regardless of their HLA binding affinities (Table 1). Interestingly, five out of eight peptides that we have characterized contained the appropriate motifs to bind both the HLA-A2 and HLA-A24 supertypes (http://www.hiv.lanl.gov/content/immunology/motif_scan/supertype.html). HLA-A2 supertype is prevalent in >40% of the world population and HLA-A24 supertype is prevalent in >50% of HBV endemic population (http://www.allelefrequencies.net/). Indeed,* in vivo* experiments using mice transgenic for the HLA-A2 or HLA-A24 molecule demonstrated that these peptides can induce T cell activation (as measured by IFN-gamma secretion) and cytolytic activity (as measured by CD107a upregulation and granzyme B secretion) in both A2 and A24 restricted manner.

Thus far, the large majority of epitope discovery for HBV has relied on peptide motif prediction software that estimates how well peptides bind to MHC class I molecules. Strong binders are selected and verified by using synthetic versions to stimulate T cells of both uninfected and infected individuals. However, peptides predicted to bind to the class I molecule may not accurately represent the epitopes presented on the surface of naturally infected cells* in vivo*, nor is a positive binding score a guarantee that T cells will be activated (unpublished observations). As such, differences in epitope identification approaches will undoubtedly lead to different immunodominant hierarchies being established. For example, Gehring et al. [40] demonstrated that, relative to other immunodominant epitopes (i.e., HBc18-27), T cell specific responses against polymerase epitopes are low. In contrast, our data indicates that, for the epitopes identified by the immunoproteomic approach, the polymerase specific T cells respond just as robustly (in terms of IFN-gamma secretion and granzyme B secretion) as those specific for core and envelope peptides.

Clearance of viral infections such as hepatitis B virus and hepatitis C virus is driven by rapid and robust CD4^+^ and CD8^+^ T cell responses. Not surprisingly, individuals who become chronically infected do not mount these vigorous responses [2, 3, 9, 41, 42]. Therefore, any therapeutic intervention to stimulate viral clearance in chronically infected patients must activate a robust, sustained T cell response directed at a diverse range of epitopes processed and presented by the chronically infected cell. A number of therapeutic vaccines for HBV have been studied in animal and human models. Although these therapeutic vaccine formulations have stimulated T cells [16–18, 43–45], sustained responses were not achieved [17, 46] and it is unclear from these studies if the T cell responses induced are capable of clearing virus from chronically infected patients. The lack of clinical responses may be due to the fact that the T cell responses induced by these therapies may not accurately reflect the epitopes that are presented by the chronically infected cells. Therefore, formulating an effective therapeutic vaccine for chronic infections should include those epitopes that are naturally processed and presented by the chronically infected cells.

Currently, there is no effective therapeutic vaccine or other immunotherapy to treat chronic HBV infected individuals. The large majority of epitope discovery by motif analysis for hepatitis B virus has been done in HLA-A2 restricted systems. Although HLA-A2 allele represents >40% of the world population, the HLA-A24 allele is more prevalent in HBV endemic population. There are no reliable algorithms for predicting HLA-A2 and A24 binding peptides. However, to design a more universal therapeutic vaccine, multiple HLA restricted epitopes need to be identified. Using an immunoproteomic approach, it is possible to purify multiple HLA alleles from the same HBV infected cell lysate and subject the isolated peptides to MS/MS analysis without a significant increase in workload or difficulty. Additionally, this approach can identify epitopes that can bind to more than one HLA allele. In our study, we identified naturally presented HBV specific T cell epitopes with double HLA binding properties, which have the potential to form the basis for a therapeutic vaccine appropriate for the HBV endemic population. Five of the identified peptides reported here share motifs that allow binding to HLA-A2 and HLA-A24 and activation of CD8^+^ T cells in both settings. Including epitopes that bind to single, distinct HLA alleles and epitopes that bind to multiple HLA alleles in a vaccine formulation may overcome HLA differences between patient populations, increase the breadth of the T cell response, and prevent escape due to antigen downregulation. These (and other) novel MHC class I binding epitopes can readily be incorporated into therapeutic vaccine formulations and tested in preclinical experiments prior to being tested in human clinical trials.

## 5. Conclusions

Our finding of novel HLA-A2 and A24-associated peptides from HBV infected cells and demonstration of HBV specific CTL responses broadens the applicability of the immunoproteomics methodology and provides candidate antigens for the development of immunotherapeutic vaccines for the treatment of chronic HBV infection. The data presented in this paper is preliminary and extensive preclinical data is needed to develop these antigens into a therapeutic vaccine.

## Supplementary Material

Identification of MHC class I restricted peptides derived from HBV that did not meet cutoff criteria. The selection criteria for peptides are as follows: Charge 1: XCorr > 1.6; Charge 2: XCorr > 1.8; Charge 3: XCorr > 2.0. Peptides that have a XCorr lower than the cutoff value are considered “low confidence” “low abundance” peptides. These peptides have been previously described in [19].

## Figures and Tables

**Table 1:** HBV specific MHC class I restricted peptides identified by immunoproteomics. The LC-MS/MS selection criteria for peptides are as follows: Charge 1: Xcorr > 1.6; Charge 2: Xcorr > 1.8; Charge 3: Xcorr > 2.0. The HLA binding scores were calculated using the SYFPEITHI prediction program [28].

Peptide sequence	Protein	Accession ID	HLA motif	*m*/*z*	Charge	Xcorr	HLA binding score
GGPNLDNIL	Large E	Q8QSF2	A2	457	2	1.88	13
LTFGRETVLEN	Precore/core (C)	Q6UFV9	A2	640	2	2.18	19
LTTVPAASLLA	Large E	Q9YKJ7	A2	530	2	2.12	20
ILRSFIPLL	Surface (S)	Q6WYY8	A2/24	537	2	1.81	A2: 28; A24: 12
FLKQQYMNL	Polymerase (P)	I0DE20	A2/24	1185	1	1.92	A2: 20; A24: 9
FLSKQYMDL	Polymerase (P)	L7QBE1	A2/A24	573	2	1.80	A2: 21; A24: 11
TVSTKLCKI	Polymerase (P)	Q8B4E6	A2/A24	497	2	1.92	A2: 19; A24: 14
FLGGPPVCL	Surface (S)	Q0EED2	A2/A24	452	2	2.05	A2: 26; A24: 11
