# Supplementary material for: MHC Class I Presented T Cell Epitopes as Potential Antigens for Therapeutic Vaccine against HBV Chronic Infection
Source: Hepat Res Treat. 2014 May 26;2014:860562. doi: 10.1155/2014/860562 (PMC4058288; doi:10.1155/2014/860562)
Supplement: Supplementary file 1 — Identification of MHC class I restricted peptides derived from HBV that did not meet cutoff criteria. The selection criteria for peptides are as follows: Charge 1: XCorr > 1.6; Charge 2: XCorr > 1.8; Charge 3: XCorr > 2.0. Peptides that have a XCorr lower than the cutoff value are considered “low confidence” “low abundance” peptides. These peptides have been previously described in [19]. [file 860562.f1.pdf]

Table 1: Identification of MHC class I restricted peptides derived from HBV that did not meet cutoff criteria. The selection criteria for peptides are as follows: Charge 1: XCorr > 1.6; Charge 2: XCorr > 1.8; Charge 3: XCorr > 2.0. Peptides that have a XCorr lower than the cutoff value are considered "low confidence" "low abundance" peptides. These peptides have been previously described [19].

Peptide sequence	Parent protein	Accession ID	Charge	XCorr
**FLPSDFFPSV**	Core protein	gi116947	2	0.59
DLLDTASALY	Core protein	gi62288052	2	1.05
EYLVSFGVW	Core protein	gi16930336	2	0.98
FLLSLGIHL	P protein	P03159	2	1.10
FLLTRILTI	Surface antigen	gi329620	2	1.02
ILLLCLIFLL	Surface antigen	gi329620	2	1.01
IPIPSSWAF	S protein	P03141	2	1.46
LPSDFFPSV	Core antigen	gi116947	2	0.70
LTFGRETVLEY	Core protein	gi16930336	2	0.69
LVVDFSQFSR	P protein	gi118870	2	0.91
LWFHISCLTF	Core protein	gi16930336	3	0.84
QAFTFSPTYK	P protein	gi399401	2	1.24
RFSWLSLLVPF	P protein	SRC7447	3	1.21
RWMCLRRFII	S protein	gi62281	2	0.69
STLPETTVVRR	Core antigen	P03146	2	1.03
WLSLLVPFV	Envelope protein	gi128168864	2	1.11
WMMWYWGPSLY	Surface antigen	gi329620	3	1.04
GLSRYVARL	P protein	gi118877	2	0.84
HTLWKAGILYK	P protein	gi118870	3	1.76
